# Knowledge, Practice, and Availability of Iodized Salt and Associated Factors in Jibat Woreda, West Shoa Zone, Ethiopia

**DOI:** 10.1155/2021/5562390

**Published:** 2021-12-23

**Authors:** Habtamu Fekadu Gemede, Badasa Tamiru, Meseret Belete Fite

**Affiliations:** ^1^Department of Food Technology and Process Engineering, Wollega University, P.O. Box 395, Nekemte, Ethiopia; ^2^Department of Food Science and Nutrition, Wollega University, P.O. Box 395, Nekemte, Ethiopia; ^3^Department of Public Health, Institute of Health Science, Wollega University, P.O. Box 395, Nekemte, Ethiopia

## Abstract

Appropriate knowledge, practice, and availability of iodized salt are used to eliminate iodine deficiency disorders. However, little is known about the availability of adequately iodized salt in the western part of Ethiopia. Thus, the aim of this study was to assess knowledge, practice, and availability of iodized salt and associated factors at household level in Jibat woreda, Ethiopia. Community-based cross-sectional study was conducted using structured and pretested questionnaire interview. Sampling salt was tested by the iodometric titration method. The result showed that iodine content more than 90% was considered as adequately iodized salt. The result of this study shown that among the 357 salt samples, 191(53.5%) households had good knowledge on iodized salt while 166 (46.5%) had poor knowledge on iodized salt. In addition, the result of the study revealed that 162 (45.4%) had good practice of iodized salt, whereas 195 (54.6%) had poor practice of iodized salt. The result of this study also shown that 149 (41.7%) households were using adequately iodized salt while 208 (58.3%) were using inadequate iodized salt in study area. Residence area, education level, household job, and average monthly income were significantly associated with knowledge of iodized salt at household level. Residence area, educational level, average monthly income, and expose to sunlight were significantly associated with availability of adequately iodized salt. In this finding, the knowledge and practices of iodized salt at household level in Jibat woreda, Ethiopia, were poor, and the availability of iodine in iodized salt was inadequate. This is associated to residence area, education level of household, and average monthly income. Therefore, any concerned body/institution should have to work in the above gabs of the knowledge, practice, and availability of iodized salt.

## 1. Background

Iodine is an essential micronutrients and dietary minerals that are needed in small amounts, for the normal physiological function of the human body. It is a critical component of thyroid hormones, which is necessary for controlling metabolic rate, growth, and development of body structures, as well as neuronal function and development. The body of an average adult person contains 20-25 mg of iodine, of which about 8 mg is present in the thyroid gland. According to the World Health Organization (WHO)/United Nations Children's Fund (UNICEF)/International Council for the Control of Iodine Deficiency Disorders (IDD) for healthy individuals, the recommended daily intake of iodine is 90 *μ*g for children 0-59 months (for less than one year), 120 *μ*g for children 6-12 years, 150 *μ*g for those aged >12 years, and 200 *μ*g for pregnant and lactating to prevent iodine deficiency disorders (IDD) [1].

Iodine exists in variable amounts in food and drinking water. Food crops lack iodine resulting in dietary iodine deficiency [2]. So, individuals require additional sources to meet the recommended amounts. When requirements are not met, thyroid hormone synthesis is impaired, resulting in hypothyroidism and a series of functional and developmental abnormalities [3, 4]. Poor intake of iodine leads to insufficient production of thyroid hormones, which affects different parts of the body, particularly muscle, control of metabolic function, reproduction, heart, liver, kidney, and the developing brain. In addition to insufficient production of thyroid hormones, iodine deficiency causes endemic goiter, cretinism, dwarfism, mental retardation, miscarriage, muscular disorders, spontaneous abortions, sterilization, and stillbirths [5].

Globally, close to 2 billion populations is at risk of iodine deficiency (ID), while one-third lives in areas where a natural source of iodine is low. In addition, IDD is more closely linked to food insecure populations, which are also often low income and educational level of household, who lack access to food, including food that may have been prepared with iodized salt. In sub-Saharan Africa, 64% of households are using iodized salt; nevertheless, the level of utilization widely varies from 10 to 90% in different countries. For instance, utilization of iodized salt is less than 10% in Sudan, Mauritanian, and Gambia, whereas in Burundi, Kenya, Uganda, and Tunisia, it is more than 90% [6]. Each year, 37 million newborns in developing countries are unprotected from lifelong causes of brain damage associated with IDD [7].

Furthermore, the level of knowledge, practice, and availability of iodized salt depends on sociodemographic characteristics (age, marital status, residence area, educational level, religion, ethnicity, households job, income); knowledge about iodized salt, practice study subject of iodized salt, and availability of iodized salt at household level in the study area. However, the actual availability of iodine in the iodized salt at the consumer level can vary over a wide range as a result of variability in the amount of iodine added during the iodization process, uneven distribution of iodine in the iodized salt, the extent of loss iodine due to salt impurities, packaging and environmental conditions during storage and transportation, loss of iodine due to washing and cooking process in the household, and the availability of noniodized salt from unconventional marketing sources [1, 4].

According to the Ethiopian Demographic and Health Survey (EDHS), only 15.4% of the households were using iodized salt. However, in Oromia region, 17% of the households were using iodized salt. Furthermore, the percentage of households that use iodized salt was generally low. Only 23.2% of urban and 13.3% of rural households were reported to have used iodized salt [8, 9]. This indicate that there is a problem related to iodized salt in the country. Therefore, this study is aimed at assessing knowledge, practice, and availability of iodized salt and associated factors at household level in Jibat woreda, Ethiopia.

## 2. Methods

### 2.1. Description of the Study Area

The study was conducted in five kebeles of Jibat woreda, which is located 184 km to the west of Addis Ababa in Western Shoa Administration Zone, Ethiopia.

### 2.2. Study Design and Period

A cross-sectional descriptive community-based survey was carried out on the knowledge and practice of iodized salt at household level by using structured and pretested questionnaire interview, and the sampling salt was tested by iodometric titration method for iodine content. The study was conducted from May, 2018 to February, 2019.

### 2.3. Study Population

All household residing in the selected kebeles by systematic random sampling techniques and those volunteered to participate in the study area.

### 2.4. Sample Size Determination

The sample size of the study was calculated using formula for estimation of single proportion [10].

(1) $$\begin{array}{c} n=\frac{{\left(\text{Z}\alpha /2\right)}^{2}\text{p }\left(1-\text{p}\right)}{d^{2}}, \end{array}$$

where *n* is the sample size, *Z* is the value corresponding to a 95%level of significant = 1.96, *P* is the expected prevalence of household availability of adequate iodized salt use (33%), *q* = 1 − *p*⟶(1 − 0.33) = 0.67, and *d* is the marginal error 5% and nonresponse rate 5%.

Therefore, from the above sample, it is  *n* = (Z*α*/2)^2^p (1 − p)/*d*^2^ = (1.96)^2^(0.33) (0.67)/(0.05)^2^ = 340.

So, with the adjustment for the nonresponse rate (5% contingency), there were *n* = 340 + 17 = 357 households.

### 2.5. Sampling Procedure

The sampling method used systematic sampling. This sampling interval was elucidated using the formula: *K* = *N*/*n* (where *K* = sampling interval by which every *K*^th^ element/subject was selected from the sampling frame. *N* = population size = 5021, *n* = sample size = 357. Hence, *K* =5021/357 = ~14). Therefore, the first household number was selected by using lottery method, and then by the systematic random sampling technique, every 14^th^ household was used to get the required number of study subjects in each kebeles.

### 2.6. Study Variables

Dependent variable is as follows: knowledge, practice, and availability of iodized salt and associated factors at household level.

Independent variables includes sociodemographic characteristics and variable question raised for knowledge about iodized salt, for practice study subject of iodized salt, and for availability of adequate iodized salt were considered as independent variables.

### 2.7. The Inclusion Criteria

All household members were participating in food item purchasing and preparation.

### 2.8. Exclusion Criteria

Individuals who were seriously ill and nonvoluntary person at the time of data collection were excluded from the study.

### 2.9. Data Collection Information

Structured questionnaires were prepared first in English language and were translated into Afan Oromo which is a local language and then translated back to English to check for consistency by doing preliminary test and pretested in Jibat woreda on 5% of the sample with the data collectors and then modified accordingly. The questionnaire was focused in the areas of the respondent sociodemographic characteristics, knowledge about iodized salt, practice of iodized salt, and availability of iodized salt at household level in the study area. Moreover, data collection and completeness of filled questionnaire were checked in the field, and the interviewers asked households to provide a teaspoon of salt used for cooking and stored in container with covered and in dry places. The salt was transported to the laboratory of Ambo University for analysis. The salt samples were analyzed quantitatively for iodine level by the idometric titration method [11]. Eight trained diploma nurses and two supervisors were needed during data collection.

### 2.10. Titration Procedure for Iodine Content in Iodized Salt Determination

10 g of salt was weighed using electronic balance and placed into a conical flask [9]. To the flask, 50 ml of water, 5 ml of 10% KI, and 1 ml of H_2_SO_4_ were all added, one by one. The solution turned a yellow/brown color, as iodine was produced. The solution was then titrated against the standardized and diluted Na_2_S_2_O_3_ until the yellow/brown color became very pale. Then, 2-3 drops of sarch indicator solution were added, which produced a dark blue-black colored complex with iodine. The titration was continued until the color completely disappears. The process was repeated to more times, and an average value for the volume of Na_2_S_2_O_3_ was determined. The concentration of iodine in salt is calculated based on the titrated volume (burette reading) of sodium thiosulphate.

### 2.11. Data Analysis

At the end of the interviews, questionnaires were checked for completeness and internal consistency. The Statistical Package for the Social Sciences (SPSS) Programme software (version 22) was used for data entry, and descriptive statistics tests were conducted for the items which were summarized by frequencies and percentages. The odds ratios (OR) at 95% confidence intervals (CI) and *p* values were obtained that is used to identify the associations' between variables.

## 3. Result

### 3.1. Sociodemographic Characteristics of the Study Participants

The sociodemographic characteristic of the respondents is shown in Table 1. Three hundred fifty seven (357) households were included in this study with a response rate of 100%. The age of study participants was 0 (0%) < 20 years, 264 (73.9%) between 20 and 40 years, and 93 (26.1%) > 40 years. More than one third 287 (80.4%) of the participant were married, 19 (5.3%) of the respondents were single, 23 (6.4%) were divorced, and 28 (7.8%) were widowed.

The study participants were predominantly Oromo, 295 (82.6%), while the rest belongs to other ethnic groups 62 (17.4%) and 204 (57.1%) were farmers, 61 (17.11%) business man, 29 (8.1%) employment, 24 (6.8%) studentsm and 39 (10.9%) others. The average monthly income of the respondents was 133 (37.3%) of the participants earned ≤100, 110 (30.8) earned between 101 and500, 46 (12.9%) earned between 501 and 999, and 68 (19.0%) earned ≥1000 in study area.

### 3.2. Knowledge of Respondents on Iodized Salt in the Study Area

More than half 194 (54.3%) of the respondents indicated that they heard about iodine while less than half 163 (45.7%) of the participants did not know what iodine means in study area (Table 2). Thirty eight 38 (10.6%) of the participants received information from radio about iodized salts, 29 (8.1%) from TV while 18 (5.0%) from books and 54 (15.1%) from health workers. Only, a relatively small 9(2.8%) and 11 (3.1%) number of respondents indicated their source as from family and friends, respectively. Nearest to half 161 (45.1%) of the participants did not heard from any sources.

The result of the study revealed that more than half 196 (54.9%) of the respondents had knowledge of iodized salts usage and 5.6%, 23.0%, 6.2%, 14.0%, and 4.2% responded as iodine that is important to prevent cretinism, prevent goiter, encouragesgood foetal growth, promote good health, and prevent dwarfism, respectively.

### 3.3. Practice of Iodized Salt Usage at Household Level in the Study Area

Table 3 shows that two thirds 236 (66.1%) of respondents were used salt for <1 week, 96 (26.9%) of them were use for 1 to 2 weeks, and 25 (7.0%) of respondents used salt for >2 weeks. Less than one fourth 87 (24.4%) of the participants were bought from shop while 270 (75.6%) of the respondents were bought from big/small market. A few 35 (2.8%) of respondents exposed the salt to sunlight.

### 3.4. Availability of Iodized Salt at Household Level in the Study Area

The availability of iodized salt at household level is shown in Table 4. More than half 237 (66.4%) of the respondents indicated their choice of salt is not readily available while 120 (33.6%) of the respondents get a choice of salts when they need, 246 (68.9%) of respondents indicated their choice of salt as being expensive ,and 111 (31.1%) of the participants indicated their choice of salt as being affordable.

The result of the study revealed that 242 (67.8%) of the participants mentioned that iodized salt did not found nearest to the home while 115 (32.2%) of the participants mentioned that iodized salts found around the home.

### 3.5. Iodine Content in Household Salt

Before testing salt samples 33 (9.20%) and 158 (44.3%) of the respondents said that they used iodized salt and uniodized salts, respectively, while the rest 166 (46.5%) of the respondents had not knew the type of salt they used.

However, after testing salt samples by using the idometric titration method among those who said they used iodized salt 14 (3.9%), those who said uniodized salt 66 (18.5%) and those who said had not knew the type of salt they used 69 (19.30%) had adequately iodized salt in study area.

### 3.6. Factors Associated with Knowledge, Practice, and Availability of Iodized Salt in the Study Area

#### 3.6.1. Factors Associated with Knowledge

Those participants who can read and write were 1.98 times more likely to have knowledge of iodized salt than those cannot read and write [AOR = 1.98, 95%CI = (1.37, 7.60)] (Table 5). The finding was supported by study done in Sodo town and Sodo Zuria woreda, Wolaita Zone, Southern Ethiopia [11].

In this study, farmers were had 1.88 times less likely to have knowledge of iodized salt than business man with [AOR = 1.88, 95%CI = (0.86, 4.08)], whereas the participants who earned average monthly income between 501_999 and ≥1000 increase the odds level of knowledge of iodized salt by 1.06 [AOR = 1.06, 95%CI = (0.04, 2.63)] and 0.86 [AOR = 0.86, 95%CI = (0.31, 2.35)] that were significantly associated with level of knowledge of iodized salt in the study area (Table 6).

The type of containers used to store salt was one of the factors associated with the good practice of iodized salt at household level. Those study participants who use container with a lid to store their salt at home were 2.60 times more likely to practice iodized salt than those who use container without cover.

Respondents with those who can read and write 1.02 times are more likely to use adequate iodized salt than those who cannot read and write (Table 7). This finding is supported by study conducted on the household use of iodized salt in Pakistan and India [12]. This might be due to the fact that education improves access and use to iodized salt.

### 3.7. Pearson's Correlation between Knowledge, Practice, and Availability of Iodized Salt

Bivariate analysis showed that level of knowledge of iodized salt scores has significantly positive correlations with practice of iodized salt (*r* = 0.41, *p* = 0.001) and availability of iodized salt (*r* = 0.12, *p* = 0.03). Additionally, practice of iodized salt at household level score has positive correlations with availability of iodized salt in the study area (*r* = 0.15, *p* = 0.004). There was a strong association between knowledge levels of iodized salt with practice of iodized salt in the study area.

## 4. Discussion

The result of the study revealed that 53.5% of the respondents had good knowledge on iodized salt while 166 (46.5%) had poor knowledge on iodized salt. A similar study done in Addis Ababa showed that 78% had good knowledge of iodized salt utilization [13]. However, the study conducted in Shebe town of South West Ethiopia showed 78.5% had poor knowledge on iodized salt [14]. This might be due to the fact that education, income, and source of information increase awareness about iodized salt and its benefits to human health and well-being. The food sources of the participants was 22 (6.2%) sea foods, 28 (7.8%) meats and its products, 23 (6.4%) iodized salt, 73 (20.4%) milk and its products, and 40 (11.2%) other sources of foods while the nearest to half 171 (47.9%) of respondents had not knew about iodine rich foods source in the study area. Majority 317 (88.8%) of the participants were stored salt in covered container while 347 (97.2%) of the respondents stored the salt in a dry place. Iodine content will remain relatively constant if the salt, kept dry, cool, and away from light [15]. The result of the study revealed that only 11 (3.1%) of the participants properly add of salts while cooking, which is the nearest to a study conducted in Burie and Womberma (West Gojam), which shows that 2% of the respondents add iodized salt at the end of cooking. Higher portion of iodine lost when salt is subjected to high temperature and heat and thus stability of iodine in salt determined by heat. Cooking loss could be a major reason for IDD [16, 17].

The result of the study revealed that 45.4% of the participants had good practice of iodized salt, whereas 195 (54.6%) had poor practice of iodized salt at household level in the study area. This finding was lower than the study done in Addis Ababa City, which shows that 76.3% of households had good practice on iodized salt but higher than the study done in Tehran, which shows that the 14% of households had good practices on iodized salt [13, 18]. Findings regarding to availability of iodized salt in the present study suggest that 41.7% of households have adequately iodized salt which was very lower than the WHO's recommendation according to which >90% of the households should utilize adequately iodized salt to eliminate IDD and in other developing countries like Kenya, Uganda, and Zimbabwe, which have successful household iodized salt coverage which is about 90% [4, 19]. However, this finding is higher than study done in Gondar, North West Ethiopia in 2012, which was 28.9%, in Asosa, 26.1%, in Bale Goba, 30%, in rural of Ada District, 39%, and DHS, 2011, which showed that 23.2% of urban households have access to iodized salt [9, 12, 20–22].

Residence of the participants was one of the factors associated with knowledge of iodized salt at household level. Accordingly, study participants who were from urban areas were 3.10 times more likely to have knowledge of iodized salt than those were from rural areas. The people in urban areas generally have more access to this type of information than those in rural areas [23]. This might be because household who lived on higher socioeconomic status had chances to purchase and use different electronic equipment which is important for enhancing nutritional education. In addition, house-to-house healthy visits by urban health workers improve knowledge of iodized salt utilization [24]. This finding is supported by study conducted in Lalo Assabi District, West Wollega Zone, Ethiopia [25], and Addis Ababa City [13] reported that the higher economic income had knowledge of iodized salt than the lower income.

Urban dwellers of participants more likely to practice iodized salt at household level than rural dwellers with [AOR = 2.77, 95%CI = (1.24, 6.20)] were significantly associated with practice of iodized salt in the study area. Findings are supported by study conducted in Malawi [26]. The odds of practicing iodized salt were 1.37 times higher among households who were can read and write compared to those who were unable to read and write. This finding was supported by studies done in Wolaita [27]. Those participants who earned average monthly income of ≥1000 birr with [AOR = 1.03, 95%CI = (0.41, 2.58)] were factors associated to practice of iodized salt in the study area. This finding was supported by study done in Asella Town Arsi Zone, Ethiopia [28]. All the type of containers used to store salt was one of the factors associated with good practice of iodized salt at household level. Those study participants who use container with a lid to store their salt at home were 2.60 times more likely to practice iodized salt than those who use container without cover.

Similar studies conducted in Jijiga town 341 (71.3%), in Ghana 62.6%, and in Neelambur, Panchayat-Coimbatore, India, 51.4% of respondents were stored salt in covered container. Loss of iodine is common in the case of the unpacked type of salt because of exposure to heat, moisture, and humidity [29–31]. In multivariate analysis, respondents from urban area were more likely to have adequately iodized salt compared to those living in the rural settings with [AOR = 2.56, 95%CI = (1.00, 4.71)]. This finding is supported by study in Lalo Assabi District, West Ethiopia, and study in Dabat District, Ethiopia, and EDHS (2011) [9, 31]. This better availability of iodized salt in the study area might be due to urban dwellers had more access to information and proximity to nearby shops in order to buy iodized salt. Respondents' monthly income is also associated with the availability of adequately iodized salt at household level. Households with the monthly income of ≥1000 increase the odds of availability level of iodized salt by 0.60 [AOR = 0.60, 95%CI = (0.66, 2.05)] that were factors associated to the availability of iodized salt. The study conducted in Ghana revealed that compared to the richest category, all other lower levels of wealth were more likely to use noniodized salt. It also shows that wealth is a significant determinant of one's likelihood of using adequately iodized salt or not [32].

A similar study conducted in Pakistan reported that income plays an important role and is the most important determinant in achieving adequate nutrition in the household [33]. This finding is also supported by study conducted in Ethiopia, in Southern Ethiopia, Sidama Zone, Bensa Woreda, and study carried in Asella town, Arsi Zone. Not exposing salt to sunlight was one of the factors significantly associated with availability of adequately iodized salt at household level in the study area. Those respondents who expose their salt to sunlight were 1.35 times less likely to practice iodized salt at household level than those who do not expose their salts in the study area.

This is consistent with the findings of the study conducted in Jijiga, Ethiopia, and Lalo Assabi district, West Ethiopia, which showed that 110 (23.0%) and 48 (6.0%) of the participants exposed the salt to sunlight, respectively. A study conducted in Delhi documented that was about 31% iodine loss from iodized salt when exposed to sunlight. This might be due to the effect of heat on the iodine content. The halogen iodide over time and exposure to excess oxygen and carbon dioxide slowly oxidizes to metal carbonate and elemental iodine which then evaporates [34].

## 5. Conclusion

The aim of this study was to assess the knowledge, practice, and availability of iodized salt and associated factors at household level in Jibat woreda, Ethiopia. The finding of the study revealed that the knowledge and practices of iodized salt at household level in Jibat woreda, Ethiopia, were poor, and the availability of iodine in iodized salt was inadequate. This is associated to residence area, education level of household, and average monthly income of the household level in the study area. Therefore, any concerned body/institution should have to work in the above gaps of knowledge, practice, and availability of iodized salt at household level in the study area. In addition, the correct storage place and use of iodized salt should be further investigated.

## Figures and Tables

**Table 1:** Frequency distribution of sociodemographic characteristics of respondents (*n* = 357).

Variables	Frequency	Percentile
Age of the participant		
<20	0	0%
20-40	264	73.9%
>40	93	26.1%
Marital status of household		
Single	19	5.4%
Married	287	80.4%
Divorced	23	6.4%
Widowed	28	7.8%
Total number of household members		
3	100	28.0%
3-5	129	36.1%
5	128	35.9%
Residence area		
Rural	275	77.0%
Urban	82	23.0%
Level of education of mother		
Cannot read and write	240	67.2%
Can read and write	55	15.4%
Grades 1-4	12	3.4%
Grade s5-8	8	2.2%
Grades 9-12	4	1.2%
Graduated	38	10.6%
Religion		
Protestant	187	52.4%
Orthodox	138	38.7%
Muslim	2	0.6%
Others	30	8.3%
What is your ethnicity?		
Oromo	295	82.6%
Amhara	46	12.9%
Tigre	2	0.6%
Gurage	13	3.6%
Kambata	1	0.3%
Household job		
Farmer	204	57.1%
Business man	61	17.1%
Employed	29	8.1%
Student	24	6.8%
Others	39	10.9%
Average monthly income		
≤ 100	133	37.3%
101_500	110	30.8%
501_999	46	12.9%
≥1000	68	19.0%

**Table 2:** Frequency distribution of respondents' knowledge about iodized salt in the study area.

Variables	Frequency	Percentile
Do you know what iodine is?		
Yes	194	54.3%
No	163	45.7%
Have you heard of a salt with chemical (iodine) added to it?		
Yes	194	54.3%
No	58	16.3%
Do not know	105	29.4%
Every salt contains iodine?		
Yes	35	9.8%
No	158	45.3%
Do not know	164	45.9%
If yes to (11), where did you hear of it?		
Radio	38	10.6%
TV	29	8.1%
Books	18	5.0%
Family	9	2.5%
Friends	11	3.1%
Health workers	54	15.1%
Others	37	10.4%
Did not heard from any sources	161	45.2%
Do you think should you take iodized salt?		
Yes	189	52.9%
No	39	10.9%
Do not know	129	36.2%
Importance of taking iodized salt		
Prevents cretinism	20	5.6%
Prevents goiter	83	23.0%
Encourages good fetal growth	22	6.2%
Promotes good health	50	14.0%
Prevents dwarfism	15	4.2%
Do not know	167	47.0%
Do any of your family members have ever suffered by ID?		
Yes	66	18.5%
No	291	81.5%
Knowledge level of household		
Good	191	53.5%
Poor	166	46.5%

**Table 3:** Frequency distribution of respondents' about practice of iodized salt in the study area.

Variable	Frequency	Percent
Which of iodine rich foods source you practice know?		
Sea foods	22	6.2%
Meats and its product	28	7.8%
Iodized salt	23	6.4%
Milk and its product	73	20.4%
Others	40	11.3%
Do not know	171	47.9%
Salt container		
Container with cover	317	88.8%
Container without cover	40	11.2%
Salt storage place		
Dry and cool place	347	97.2%
Moisture/heat area	10	2.8%
Washing salt before use		
Yes	71	19.9%
No	286	80.1%
Types of salt do you use know		
Iodized salt only	33	9.2%
Uniodized salt	158	44.3%
Do not know	166	46.5%
Where do you usually purchase salt?		
Shop	87	24.4%
Big/small market	270	75.6%
Period of use (weeks)		
<1 week	236	66.1%
1-2 weeks	96	26.9%
> 2 weeks	25	7.0%
Expose to sunlight?		
Yes	10	2.8%
No	347	97.2%
Practice (add) of salt while cooking		
In the beginning	115	32.2%
Halfway through cooking	206	57.7%
After cooking	11	3.1%
Towards the end	25	7.0%
Taste difference between iodized salt and salt no chemical (iodine) added?		
Yes	67	18.8%
No	61	17.1%
Do not know	229	64.1%
Practice level of participant		
Good	162	45.4%
Poor	195	54.6%

**Table 4:** Frequency distribution of respondent's availability of iodized salt in the study area.

Variables	Frequency	Percentile
Do you easily get your choice of salt when you need it?		
Yes	120	33.6%
No	237	66.4%
Price of your choice of salt		
Expensive	246	68.9%
Affordable	111	31.1%
Iodine salt is found nearest to your home?		
Yes	115	32.2%
No	242	67.8%
Availability level of iodine content in salt		
Adequate	149	41.7%
Inadequate	208	58.3%

**Table 5:** Factors associated with knowledge of iodized salt in the study area.

Variables	Frequency	Percent	Crude OR (95% CI)	Adjusted OR (95% CI)
Area				
Rural	275	77.0%	1	1
Urban	82	23.0%	6.66 (3.52, 12.61)^∗^	3.10 (0.76, 4.31)^∗^
Education of household				
Cannot read and write	240	67.2%	1	1
Can read and write	55	15.4%	2.10 (3.25, 11.18)^∗∗^	1.98 (1.37, 7.60)^∗^
Grades 1-4	12	3.4%	4.74 (1.61, 14.00)	1.27 (1.71, 9.52)
Grades 5-8	8	2.2%	1.32 (2.87, 6.10)	1.21 (2.79, 5.26)
Grades 9-12	4	1.1%	1.98 (3.10, 12.67)^∗^	1
Graduated	38	10.6%	3.10 (2.05, 7.02)^∗^	1
Household job				
Farmer	204	57.1%	1	1
Business man	61	17.1%	2.37 (1.18, 4.77)^∗∗^	1.88 (0.86, 4.08)^∗^
Employed	29	8.1%	0.76 (0.33, 1.73)	0.82 (0.35, 1.95)
Student	24	6.7%	1	1
Others	39	19%	0.13 (0.27, 0.64)^∗∗^	0.12 (0.02, 0.95)
Average monthly income				
≤ 100	133	37.3%	1	1
101_500	110	30.8%	6.39 (3.18, 12.82)^∗∗^	1.58 (0.64, 3.89)
501_ 999	46	12.9%	4.08 (2.00, 8.30)^∗∗^	1.06 (0.04, 2.63)^∗^
≥1000	68	19.0%	2.10 (1.28, 6.91)^∗^	0.86 (0.31, 2.35)^∗^
Salt container				
Container with cover	317	88.8%	2.08 (1.05, 4.10)^∗^	1.36 (0.65, 2.87)
Container without cover	40	11.2%	1	1
Washing salt before use				
Yes	71	19.9%	1	1
No	286	80.1%	2.93 (2.05, 5.22)^∗^	1.83 (1.75, 2.77)
Expose to sunlight?				
Yes	10	2.8%	1	1
No	347	97.2%	0.45 (0.27, 0.76)^∗^	0.30 (0.04, 0.57)

**Table 6:** Factors associated with practice of iodized salt in the study area.

Variables	Frequency	Percent	Crude OR (95% CI)	Adjusted OR (95% CI)
Residence area				
Rural	275	77.0%	1	1
Urban	82	23.0%	3.43 (2.03, 5.80)^∗^	2.77 (1.24, 6.20)∗
Educational level of mother				
Cannot read and write	240	67.2%	1	1
Can read and write	55	15.4%	3.55 (1.71, 7.38)^∗^	1.37 (0.47, 3.97)^∗^
Grades 1-4	12	3.4%	2.09 (0.88, 4.96)	1.11 (0.39, 3.13)
Grades 5-8	8	2.2%	0.72 (0.17, 3.16)	0.28 (0.05, 1.45)
Grades 9-12	4	1.1%	2.17 (0.46, 10.16)	1.04 (0.90, 5.68)
Graduated	38	10.6%	2.89 (1.94, 5.21)	1
Average monthly income				
≤100	133	37.3%	1	1
101_500	110	30.8%	2.60 (1.42, 4.73)	1.08 (0.47, 2.47).
501_ 999	46	12.9%	2.09 (1.31, 3.87)^∗∗^	0.91 (0.40, 2.10)
≥1000	68	19.0%	1.92 (0.90, 4.11)^∗∗^	1.03 (0.41, 2.58)^∗^
Container of salt				
Container with cover	317	88.8%	3.81 (1.04,4.30)^∗∗^	2.60 (1.83, 3.50)^∗^
Container without cover	40	11.2%	1	1
Washing salt before use				
Yes	71	19.9%	1	1
No	286	80.1%	2.32 (1.32, 4.10)^∗∗^	0.20 (0.08, 0.36)
Expose to sunlight?				
Yes	10	2.8%	1	1
No	347	97.2%	3.19 (1.89, 6.51)^∗^	1.75 (0.89, 3.21)

**Table 7:** Factors associated with availability of iodized salt in the study area.

Variables	Frequency	Percent	Crude OR (95% CI)	Adjusted OR (95% CI)
Residence area				
Rural	275	77.0%	1	1
Urban	82	23.0%	3.27 (1.39, 7.93)^∗^	2.56 (1.00, 4.71)^∗^
Education of household				
Cannot read and write	240	67.2%	1	1
Can read and write	55	15.4%	13.10 (5.25, 37.18)^∗∗^	1.02 (1.37, 7.60)^∗^
Grades 1-4	12	3.4%	4.74 (1.61, 14.00)	1.27 (1.71, 9.52)
Grades 5-8	8	2.2%	1.32 (2.87, 6.10)^∗∗^	1.21 (2.79, 5.26)
Grades 9-12	4	1.1%	1.98 (3.10, 12.67)^∗^	1
Graduated	38	10.6%	3.74 (2.91, 9.65)^∗^	1
Household jobs				
Farmer	204	57.1%	1	1
Business man	61	17.1%	3.53 (1.74, 7.16)	0.86 (0.34, 2.19)
Employed	29	8.1%	0.50 (0.44, 2.26)^∗∗^	0.93 (0.300, 2.88)
Student	24	6.7%	0.30 (0.94, 1.70)^∗^	
Others	39	19%	2.01 (0.72, 5.70)	
Average monthly income				
≤100	133	37.3%	1	1
101_500	110	30.8%	7.67 (3.88, 15.11)	0.63 (0.22, 1.84)
501_ 999	46	12.9%	7.26 (3.61, 14.60)^∗∗^	0.58 (0.19, 1.76)
≥1000	68	19.0%	4.21 (1.86, 9.51)^∗∗^	0.60 (0.66, 2.05)^∗^
Salt container				
Container with cover	317	88.8%	0.31 (0.14, 0.70)^∗^	1.52 (0.66, 3.50)
Container without cover	40	11.2%	1	1
Expose to sunlight?				
Yes	10	2.8%	1	1
No	347	97.2%	2.19 (1.89, 6.51)^∗^	1.35 (0.69, 3.01)^∗^

## Data Availability

All the data generated from this study have been presented in the manuscript
